# Prescreening for Osteoporosis With Quantitative Ultrasound in Postmenopausal White Women

**DOI:** 10.1002/jum.14844

**Published:** 2018-10-20

**Authors:** Bernhard Steiner, Hans Peter Dimai, Hubert Steiner, Sabrina Cirar, Astrid Fahrleitner‐Pammer

**Affiliations:** ^1^ Department of Internal Medicine, Division of Endocrinology and Diabetology Medical University of Graz Graz Austria; ^2^ OG‐Ost Apparategemeinschaft Osteoporose St Peter Graz Austria

**Keywords:** bone mineral density, dual‐energy x‐ray absorptiometry, osteoporosis, postmenopausal women, prescreening, quantitative ultrasound

## Abstract

**Objectives:**

Calcaneal quantitative ultrasound (QUS) is a readily accessible and radiation‐free alternative to dual‐energy x‐ray absorptiometry (DXA) for assessing bone mineral density (BMD). Results obtained from QUS measurement cannot directly be compared to DXA, since these techniques capture different bone‐specific parameters. To identify individuals who are likely to have osteoporosis by DXA, device‐specific thresholds have to be defined for QUS. This cross‐sectional study evaluated the accuracy of QUS to identify postmenopausal women with osteoporosis, defined as a T score of –2.5 SDs or lower by DXA, and to calculate device‐specific cutoff values for the QUS device investigated.

**Methods:**

We assessed BMD at the lumbar spine, bilateral femoral neck, and total hip sites with DXA and QUS parameters of the right and left calcanei in a cohort of 245 postmenopausal treatment‐naïve women between 40 and 82 years. Correlation coefficients for BMD and QUS parameters were calculated. Receiver operating characteristic curves were generated, and areas under the curves (AUCs) were evaluated. Cutoff values for QUS were defined.

**Results:**

Calcaneal QUS had the ability to identify postmenopausal women with a T score of –2.5 or lower at the right hip (AUC, 0.887) and left femoral neck (AUC, 0.824). Cutoff values for the QUS T scores at the right (–1.455) and left (–1.480) calcanei were defined for screening purposes.

**Conclusions:**

This study provides insights into the comparative performance of QUS with DXA. Considering the diagnostic accuracy of this modality in comparison to DXA, it can be recommended as a prescreening tool to reduce the number of DXA screenings.

**Abbreviations**
AUC: area under the curve
BMD: bone mineral density
BMI: body mass index
BUA: broadband ultrasound attenuation
DXA: dual‐energy x‐ray absorptiometry
QUS: quantitative ultrasound
SOS: speed of sound

Due to aging societies around the world, osteoporosis and osteoporotic fractures have become major global burdens for health care providers and health care systems. The economic burden of osteoporotic fractures has been highlighted in previous studies. In 2010, Europe's 6 largest countries expended €31 billion for the treatment of osteoporotic fractures.^1^ The lifetime risks of a fragility fracture are approximately 50% for women and 25% for men older than 50 years, and these rates are likely to rise in the near future because of demographic changes.^2^

Current Austrian practice guidelines, as published recently by the Main Association of Austrian Social Security Institutions, advocate the assessment of fracture risk by using the fracture risk assessment tool FRAX (University of Sheffield, Sheffield, England), which aside from clinical risk factors also takes into account the bone mineral density (BMD) of the femoral neck.^3^ However, this approach is limited on the one hand by the availability of dual‐energy x‐ray absorptiometry (DXA) devices and on the other hand by a widespread lack of awareness and perception of osteoporosis not only in the general population but also among health care professionals.^4^, ^5^, ^6^

According to the World Health Organization, osteoporosis has been defined as a bone mineral density of –2.5 SDs or greater below the average value for young healthy individuals, described as a T score of –2.5 SDs or lower, as measured with DXA.^7^ On the basis of this definition, quantitative ultrasound (QUS) cannot be used as a tool to diagnose osteoporosis, since bone‐specific parameters as captured by QUS are different from those captured by DXA.

However, QUS of the calcaneus has been shown to be a useful means for early diagnosis of and prescreening for osteoporosis.^8^, ^9^, ^10^, ^11^ Compared to DXA, which is center bound and emits low doses of ionizing radiation, QUS is transportable, inexpensive, time saving, and radiation free.^4^ Previous studies suggested that QUS of the calcaneus could be used as a prescreening tool to identify patients at high risk, for whom treatment should be initiated, just as it could limit the number of patients who need a DXA measurement by predicting patients at very low risk.^12^, ^13^ As a result of differing measurement techniques and skeletal sites, cutoff values vary between different QUS devices. The purpose of this study was to identify optimal cutoff values for a specific calcaneal QUS device (Pegasus; Medilink, Mauguio, France) to identify patients at risk of osteoporosis in postmenopausal women.

## Materials and Methods

### *Participants*

This cross‐sectional study was performed at a private practice specializing in osteoporosis screening (OG‐Ost Apparategemeinschaft Osteoporose St Peter). Between May and July 2017, postmenopausal white women, aged 40 to 82 years, who were untreated and had no history of secondary osteoporosis, were invited to have a DXA scan performed together with calcaneal QUS measurement.

Exclusion criteria were treatments known to affect bone metabolism, except calcium and vitamin D, previous diagnosis of osteoporosis. and any case of secondary osteoporosis. Ethical approval for the study protocol was received by the Institutional Review Board of the Medical University of Graz (30‐229 ex 17/18).

### *Measurements*

All DXA measurements were conducted with the same device (Lunar Prodigy Pro; GE Healthcare, Buc, France), with least significant changes of 0.021 g/cm² at the left total hip and 0.027 g/cm² at the right total hip. Values for the BMD, T score, and Z score at the lumbar spine (at least 2 assessable vertebrae), left and right femoral neck regions, and total hip region, as well as trabecular bone structure were assessed. Based on the World Health Organization classification, a T score of –2.5 or lower was categorized as osteoporosis, a T score higher than –2.5 to lower than –1.0 as osteopenia, and a T score higher than –1.0 as normal.

Quantitative US measurements were performed on the same day as the DXA measurements, using a single unit of the gel‐coupled Pegasus QUS device. Values measured and calculated by this device include broadband ultrasound attenuation (BUA; decibels per megahertz), speed of sound (SOS; meters per second), QUS T score, which is equivalent to the DXA T score, and QUS Z score, which is equivalent to the DXA Z score.

A wall‐mounted standardized stadiometer was used to determine height to the nearest centimeter, and body weight was measured on a calibrated digital scale noted to the nearest 0.1 kg. All measurements were performed by a single certified well‐trained medical technician, and results were entered into a password‐protected anonymized database.

### *Statistical Analysis of the Study Population*

All variables were checked for plausibility to detect outliers in the data set. The assumption of a normal distribution was proven by Shapiro‐Wilk tests (*P* > .05 normally distributed data assumed) and Q‐Q plots. Descriptive statistics were calculated for continuous variables, which are presented as means and standard deviations. To analyze associations of DXA (T score for L1–L4, including trabecular bone structure, left and right femoral neck regions, and total hip) with QUS (BUA, SOS, and QUS T score) and the body mass index (BMI), Pearson correlation coefficients were computed for normally distributed data; otherwise, the Spearman coefficient was applied. A receiver operating characteristic curve analysis was used to evaluate the discriminatory ability of QUS to detect postmenopausal women with and without osteoporosis as defined by the reference standard DXA (T score of –2.5 or lower). The area under the curve (AUC) was calculated for available DXA sites with a confidence interval of 95%. Describing the inherent validity of diagnostic tests, the AUC is an effective and combined measure of sensitivity and specificity.^14^ The sensitivity (percent) and specificity (percent) were determined at various cutoffs with a DXA T score of –2.5 or lower for L1–L4, the right and left femoral neck regions, and total hip combined into a single variable to evaluate the overall capability of this QUS device to diagnose osteoporosis in the studied cohort. This process was done by combining all cases with at least a single DXA T score of –2.5 or lower into a single variable. SPSS version 22.0 software (IBM Corporation, Armonk, NY) and Stata version 12 software (StataCorp, College Station, TX) were used for the statistical analysis. Two‐tailed *P* < .05 was considered statistically significant.

## Results

A total of 245 participants was screened for eligibility. Eleven patients had to be excluded because of invalid measurements; thus, 234 cases were included in the final analysis.

The characteristics of the study population are summarized in Table 1. The T score at the lumbar spine ranged from –4.9 to 2.6 SDs with a mean –1 ± 1.4 SDs. With a mean BMI of 24.98 ± 4.47 kg/m^2^, the population investigated was near the upper threshold of normal weight. Women with osteoporosis had a lower BMI, T score, BUA, and QUS T score (*P* < .001) compared to women without osteoporosis. A total of 165 participants were between 50 and 65 years. Twenty‐four of the 32 cases with a DXA T score of –2.5 or lower were found in this cohort. The mean age of this group was 56.67 years, and the mean BMI was 24.3 kg/m^2^.

**Table 1:** Descriptive Characteristics and Results of DXA and QUS Measurements of the Study Population

Characteristic	All Women			Women Without Osteoporosis			Women With osteoporosis		
	n	Mean or %	SD	n	Mean or %	SD	N	Mean or %	SD
Age, y	234	59.75	8.476	202	59.2	8.3	32	62	8.4
40–49	15	6.4%		15	6.4%		0	0%	
50–59	123	52.1%		106	45.3%		17	7.3%	
60–69	58	24.6%		49	20.9%		8	3.4%	
70–82	40	16.7%		32	13.7%		7	3.0%	
Body mass, kg	236	66.4	11.6	202	67.6	11.4	32	58.1	9.7
BMI, kg/m^2^	236	24.9	4.4	202	25.4	4.3	32	22.3	3.3
DXA T score	234								
Normal	106	45.3%							
Osteopenia	96	41.0%							
Osteoporosis	32	13.7%							
DXA									
T score L1–L4	234	−1.0	1.4	198	−0.7	1.1	32	−3.2	0.6
T score hip (left)	233	−0.83	1.1	195	−0.7	1.1	32	−1.9	0.8
T score hip (right)	229	−0.8	1.1	192	−0.6	1.1	31	−1.9	0.9
Trabecular bone structure	231	1.3	0.1	195	1.3	0.1	32	1.2	0.1
QUS									
BUA (left)	232	67.8	7.46	198	68.5	7.4	32	63.0	6.6
T score (left)	231	−0.5	1.16	197	−0.4	1.1	32	−1.3	1.0
SOS (left)	232	1417.3	27.38	198	1419.2	27.1	32	1405.2	27.1
BUA (right)	230	68.4	6.77	197	69.0	6.6	31	64.3	6.2
T score (right)	230	−0.39	1.05	197	−0.3	1.0	31	−1.0	0.99
SOS (right)	230	1439.1	192.769	197	1431.8	123.6	31	1486.9	427.4

The following bivariate correlations were all significant at the .01 level. A moderate positive correlation of DXA and QUS measurements was evident between the T score at the left hip and QUS T score at the left calcaneus (*r* = 0.515). There was a weaker correlation between the QUS T score at the left calcaneus and the T score at the lumbar spine (*r* = 0.397). The QUS T score at the right calcaneus had slightly lower correlations with the DXA T score at the right hip (*r* = 0.505) and lumbar spine (*r* = 0.404). A strong positive correlation was noted between the BUA measurements at the right and left calcaneus (*r* = 0.834), whereas the SOS at the left calcaneus moderately correlated with left BUA measurements (*r* = 0.541). There was no significant correlation between the SOS and BUA at the right calcaneus. A weak but significant positive correlation was noted between the trabecular bone structure and left (*r* = 0.309) as well as right (*r* = 0.333) BUA. The BMI had very weak positive significant correlations with SOS measurements of the right (*r* = 0.218) and left (*r* = 0.207) sides.

Since some of the studied variables did not follow a normal distribution, nonparametric estimation AUCs from the receiver operating characteristic curve were computed. The accuracy of QUS to detect women with osteoporosis at the lumbar spine, left and right hips, and femoral neck is summarized in Tables 2 and 3. Better overall performance was associated with an AUC of greater than 0.80. High diagnostic accuracy was achieved at the right hip between the DXA T score and the QUS T score at the right calcaneus (AUC, 0.887). At the right hip, no significant difference could be found between the QUS T score at the right calcaneus and the QUS T score at the left calcaneus (*P* = .093). At the left calcaneus, results for the left femoral neck (AUC, 0.823) outperformed those for the left hip (AUC, 0.772). The diagnostic accuracy at the lumbar spine, paired with calcaneus measurements of the right (AUC, 0.700) and left (AUC, 0.704) sides, was slightly lower. When QUS T scores were paired with the combined findings of all DXA sites, the right (AUC, 0.732) and left (AUC, 0.731) QUS measurements achieved nearly the same AUCs. The best AUC for isolated DXA sites was calculated for participants between 50 and 65 years for the right (AUC, 0.956) and left (AUC, 0.95) calcanei when paired with DXA results for the ipsilateral hip.

**Table 2:** Receiver Operating Characteristic Curve Analysis to Identify DXA T Scores of –2.5 or Lower at the Right Calcaneus

Parameter	AUC	SD	*P*	95% CI
At the right femoral neck: 7 positive, 221 negative (n = 228)				
BUA	0.770	0.057	.015	0.658–0.882
QUS T score	0.736	0.071	.033	0.596–0.876
SOS	0.619	0.134	.283	0.357–0.882
At the right hip: 10 positive, 219 negative (n = 229)				
BUA	0.885	0.034	<.001	0.819–0.951
QUS T score	0.887	0.033	<.001	0.822–0.952
SOS	0.714	0.088	.022	0.541–0.886
At the lumbar spine: 31 positive, 197 negative (n = 228)				
BUA	0.708	0.049	<.001	0.611–0.805
QUS T score	0.700	0.050	<.001	0.603–0.798
SOS	0.602	0.051	.069	0.503–0.701

CI indicates confidence interval.

**Table 3:** Receiver Operating Characteristic Curve Analysis to Identify DXA T Scores of –2.5 or Lower at the Left Calcaneus

Parameter	AUC	SD	*P*	95% CI
At the left femoral neck: 11 positive, 220 negative (n = 231)				
BUA	0.810	0.054	.001	0.703–0.916
QUS T score	0.824	0.053	<.001	0.720–0.929
SOS	0.717	0.078	.015	0.564–0.871
At the left hip: 14 positive, 217 negative (n = 231)				
BUA	0.758	0.058	.001	0.644–0.871
QUS T score	0.772	0.057	.001	0.660–0.884
SOS	0.698	0.062	.013	0.578–0.819
At the lumbar spine: 32 positive, 197 negative (n = 229)				
BUA	0.698	0.048	<.001	0.603–0.793
QUS T score	0.704	0.048	<.001	0.609–0.799
SOS	0.621	0.048	.028	0.528–0.714

CI indicates confidence interval.

Table 4 summarizes the performance of QUS to determine postmenopausal women with osteoporosis at suitable cutoff points. Cutoffs of –1.455 for the right and –1.48 for the left QUS T scores achieved acceptable specificities to qualify as screening parameters for osteoporosis. Separate cutoffs were defined for women between 50 and 65 years. For the left calcaneus, a QUS T score of –1.325 resulted in 62.5% sensitivity and 83.1% specificity. At the right calcaneus, a QUS T score of –1.305 achieved 54.2% sensitivity and 86% specificity.

**Table 4:** Sensitivity and Specificity at Suitable Cutoffs to Identify DXA T Scores of –2.5 or Lower

Parameter	Cutoff	Sensitivity, %	Specificity, %
QUS T score (right)	−0.125	78.5	48.9
	−0.780	69.2	68.8
	−1.455	41.0	86.6
QUS T score (left)	−0.235	79.5	44.6
	−0.800	66.7	67.2
	−1.480	51.3	83.3

## Discussion

With a hip fracture incidence of less than 650 per 100,000 in a population aged 50 years or older, Austria has the third highest rate of hip fractures in the European Union.^15^ Especially in people older than 85 years, adequate assessment of osteoporosis is lacking, and the prevalence of osteoporosis is higher than the documented diagnosis.^16^ Screening with DXA is most commonly used in patients who already have had their first fragility fracture to initiate osteoporosis treatment. However, prevention of the first fragility fracture should be the primary objective to reduce the burden of disability, increased costs, and increased mortality risk inflicted by fragility fractures. However, widespread DXA screening for osteoporosis in the whole population is neither recommended nor accomplishable.^17^ To prevent the first fragility fracture, a prescreening method appears to be useful for identifying individuals who are at high risk of osteoporosis and osteoporotic fractures. At the same time, prescreening with QUS may reduce the number of unnecessary DXA measurements in individuals with high BMD and consequently a lower fracture risk.^17^ In this study, QUS of the calcaneus was shown to be an attractive method for osteoporosis prescreening because of its portability, low cost, and easy usability. Quantitative US devices such as the one investigated in this study can easily be taken to rural areas or to patients who are home bound and can be deployed by trained staff in pharmacies or physicians’ offices to screen for suspected osteoporosis.^18^

In the population of postmenopausal women investigated in this study, a significant correlation was found between each DXA site and the QUS parameters. Consistent with other studies among older women, the correlations between the QUS T score and DXA T score at the hip and femoral neck were better than those for the lumbar spine T scores.^17^, ^19^

The ability of calcaneal QUS to identify women with a DXA T score of –2.5 or lower at the right hip (AUC, 0.887) excelled in comparison to a T score of –2.5 or lower at the lumbar spine (AUC, 0.704) or left femoral neck (AUC, 0.824). Additionally, noteworthy was the very strong AUC of 0.956 for measurements of the right calcaneus and right hip in patients between 50 and 65 years, which would indicate an especially high potential use of this device for the prescreening of hip fractures in women of this age group. The sensitivity and specificity of the calculated cutoff values were too low for the diagnosis of osteoporosis. Device‐specific cutoff values with acceptable specificity were defined for the screening of osteoporosis. For the left calcaneus, a QUS T score of –1.480 was identified, and for the right calcaneus, a QUS T score of –1.455 was identified. For measurement results below the defined cutoff values, additional DXA screening would be advisable, and patients with scores above these cutoff values would be considered to be at low risk. The performance of the investigated device is comparable with well‐studied QUS devices. Boonen et al^20^ did achieve overall sensitivity of 68% and specificity of 70% (AUC, 0.72) using Sahara equipment (Hologic, Marlborough, MA) in a community‐dwelling population of postmenopausal women (n = 221). Gemalmaz et al^21^ studied a total group of 919, including 87 men, using a GE Lunar Achilles Express device, resulting in overall sensitivity of 73.7% and specificity of 57.4%. Larijani et al^22^ deployed the Achilles device, investigating a population of 420 postmenopausal women, resulting in total sensitivity of 84.7% and specificity of 50%. Flöter et al^23^ reviewed 6 articles that compared QUS of the calcaneus to DXA as the reference standard. As in this study, thresholds were chosen for QUS T score but with a variability of –1.7 to –2.4 lower as the chosen values for the device investigated in this study.^23^ In these population‐based studies the crucial point is the determination of a device‐ and population‐specific cutoff point, which explains the differences in specificity and sensitivity. Not only the device used but also the population investigated is a factor that results in different cutoff values. This necessity to define specific cutoff values for each device and population is likely to limit the usefulness of QUS as a screening tool. Our study only included white postmenopausal untreated women with higher educational backgrounds. However, our results for this device are comparable to results from different populations and other devices,^10^ a fact that underlines the potential of QUS as an effective screening tool.

This study had several limitations, including a possible cohort effect; since all patients were referred to the office for osteoporosis screening, a selection bias occurred. Unlike a real‐life study, which would have had a broad spectrum of patients, this study took place at a private institute and thus resulted in a filtered group of participants who were mainly well educated and consisted solely of postmenopausal women older than 40 years.

In conclusion, this study provides information on the usefulness of a specific calcaneal QUS device as an osteoporosis screening tool in postmenopausal women. The comparative performance of the investigated QUS device with DXA was assessed. Due to its low sensitivity, this QUS device cannot be recommended for diagnosis of osteoporosis as defined by the World Health Organization. As shown for other QUS devices before, the predictive value of the QUS device tested in the prevailing study for BMD is low.^22^, ^24^ However, given the cutoff values’ specificity of 86.6% at the right calcaneus and 83.3% at the left calcaneus, this QUS device can be recommended as a prescreening tool to decide whether a DXA measurement should be performed. Future studies in a larger cohort of postmenopausal women representative of the general population are needed to further support these findings.
